# *Ex Vivo* Analysis of Human Memory B Lymphocytes Specific for A and B Influenza Hemagglutinin by Polychromatic Flow-Cytometry

**DOI:** 10.1371/journal.pone.0070620

**Published:** 2013-08-15

**Authors:** Monia Bardelli, Liliana Alleri, Francesca Angiolini, Francesca Buricchi, Simona Tavarini, Chiara Sammicheli, Sandra Nuti, Elena Degl'Innocenti, Isabelle Isnardi, Elena Fragapane, Giuseppe Del Giudice, Flora Castellino, Grazia Galli

**Affiliations:** 1 Novartis Vaccines and Diagnostics srl, Siena, Italy; 2 Novartis Institutes for Biomedical Research, Basel, Switzerland; University of Georgia, United States of America

## Abstract

Understanding the impact that human memory B-cells (MBC), primed by previous infections or vaccination, exert on neutralizing antibody responses against drifted influenza hemagglutinin (HA) is key to design best protective vaccines. A major obstacle to these studies is the lack of practical tools to analyze HA-specific MBCs in human PBMCs *ex vivo*. We report here an efficient method to identify MBCs carrying HA-specific BCR in frozen PBMC samples. By using fluorochrome-tagged recombinant HA baits, and vaccine antigens from mismatched influenza strains to block BCR-independent binding, we developed a protocol suitable for quantitative, functional and molecular analysis of single MBCs specific for HA from up to two different influenza strains in the same tube. This approach will permit to identify the naive and MBC precursors of plasmablasts and novel MBCs appearing in the blood following infection or vaccination, thus clarifying the actual contribution of pre-existing MBCs in antibody responses against novel influenza viruses. Finally, this protocol can allow applying high throughput deep sequencing to analyze changes in the repertoire of HA^+^ B-cells in longitudinal samples from large cohorts of vaccinees and infected subjects with the ultimate goal of understanding the *in vivo* B-cell dynamics driving the evolution of broadly cross-protective antibody responses.

## Introduction

The surface glycoprotein hemagglutinin (HA) plays a critical role in influenza virus infection, by anchoring viruses to surface sialic-acid residues on host cells and by mediating the subsequent fusion of viral and host cell membranes. Antibodies blocking these interactions are the only widely recognized correlate of protection from infection. Both influenza infection and vaccination prime durable immune memory in humans [1]–[3]. Priming of immune memory by overt or subclinical influenza infection can occur early in life, thus most human immunizations occur in the context of pre-existing immunity. Influenza HA is highly susceptible to mutations and drifted variants capable to escape pre-existing neutralizing antibodies emerge continuously. For this reason influenza vaccines must be reformulated yearly. Whether, and to what extent, pre-existing memory B-cells (MBCs) play a role in preventing infection by new influenza variants is poorly understood [4]–[5].

Convincing evidence showing that MBCs are recruited in early plasmablast responses to infection or vaccination has been collected by several groups [6]–[10], also during the 2009 pandemic [10]–[12]. Most of this information has been obtained by applying the best state-of-the-art technologies for molecular cloning and expression of paired heavy and light variable immunoglobulin (IgV_H_V_L_) genes to arrays of single plasmablasts from multiple subjects [6], [8]–[11]. This has been possible because plasmablasts are identifiable by flow-cytometry based on the expression of well-defined surface markers but mostly because they appear in large numbers in the blood one week following infection or vaccination and therefore don't need to be selected based on antigen specificity [6]. Applying similar approaches to analyze the repertoire of pre-existing antigen specific-MBCs would be key to verify their actual contribution in plasmablast responses to drifted HA antigens, as well as in antigen-driven germinal center reactions that ultimately generate long-lived antibody secreting cells and memory B-cells expressing antibodies of refined specificities. A major obstacle to move in this direction is the lack of practical markers to identify rare antigen-specific MBCs within the bulk of MBCs present in *ex vivo* human PBMCs. Successful attempts to analyze and sort by flow-cytometry mouse B-cells binding to fluorochrome-labeled soluble HA molecules have been reported several years ago [13]. Unfortunately, applying similar approaches to the analysis of PBMC samples from human influenza patients or vaccinees has proved challenging so far [14]–[15], due to non-specific binding of HA to the surface of all human leukocytes.

We explored different approaches to sort HA-specific MBCs and found that an efficient method to prevent non specific binding of influenza HA is pre-saturation of PBMCs with influenza mono-bulk vaccine antigens (that is, monovalent bulk vaccine antigen before final formulation into multivalent mixtures, filling, and finishing) from a strain mismatched to the one used as fluorescent bait. By using influenza A and B mono-bulks as saturating reagents, we developed a staining protocol suitable for direct flow-cytometric analysis of B-cells specific for HA from up to two different mismatched influenza strains in the same human PBMCs sample. This technique can be applied to monitor quantitative and qualitative changes in the distribution of HA binding across different B-cell subsets following vaccination, and to obtain enriched population of HA-specific B-cells for molecular cloning of paired V_H_V_L_-Ig genes. This protocol provides a unique tool to compare HA-specific B-cell repertoires across cohorts of subjects with different histories of influenza exposure and to obtain information suitable for the development of novel influenza vaccines.

## Results

### Detection of BCR-dependent binding to soluble influenza recombinant HA baits

**Figure 1:**
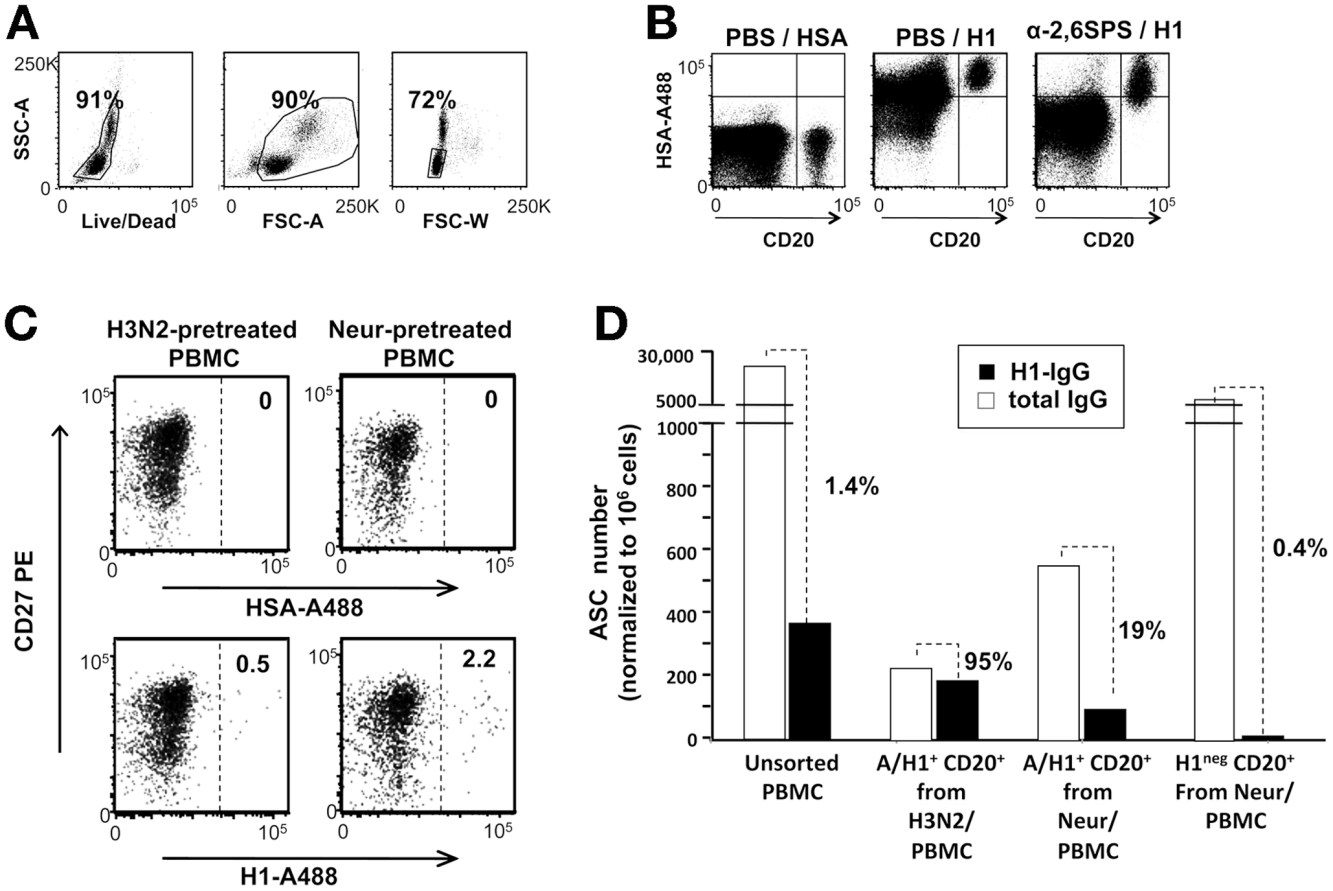
Blockade of sialic-acid binding sites reveals BcR-dependent binding to influenza HA. **A–B.** Diffuse binding of rH1 to untreated human PBMC. Thawed PBMCs from an anonymous blood donor were first stained with Live/Dead then with an anti-CD20 mAb mixed with Alexa488-conjugated human serum albumin (PBS/HSA), or Alexa488-conjugated rH1 from the A/California/07/2009 strain (PBS/rH1), or a solution of sialopentasaccarides containing the Alexa488-conjugated rH1 (α-2,6-SPS/H1Cal). The strategy for gating on single live lymphocytes is shown in A; rH1 binding to CD20 negative (CD20^neg^) and CD20^+^ cells is shown in B. **C.** Blockade of sialic-acid binding sites reveals putative BcR-specifc binding of rH1. PBMCs from a single donor were split in 8 tubes (10^7^ PBMC/each), 4 tubes were incubated with H3N2 mono-bulk subunit vaccine antigens from the A/Panama/2007/1999 strain (left dot plots), and 4 tubes with neuraminidase (right dot plots). Then the PBMCs were incubated with anti-CD20 and anti-CD27 mAbs and A488-HSA (upper plots), or A488-rH1 (lower plots). Shown is the distribution of rH1 binding on memory (CD27^+^) and naïve (CD27^neg^) B cells identified in the CD20^+^ gate. **D.** Specificity of rH1 binding. H1^+^ B-cells sorted from H3N2- (n = 1372) or neuraminidase- (Neur) (n = 2233) treated PBMCs were mixed with sorted autologous CD20^−^ cells in the ratio of 1∶200 and activated in vitro with CpG and IL-2 for 5 days. As controls, cultures of unsorted PBMCs, as well as of autologous CD20^neg^ cells mixed with H1-negative (H1^neg^) B cells sorted from neuraminidase-treated PBMCs (n = 30,000) in the ratio of 1∶10 were run for 5 days in the presence of CpG and IL-2. After 5 days, cells from each culture were harvested, washed, counted and assayed by ELISPOT to enumerate cells secreting IgG and H1N1-specific IgG. To facilitate comparisons between different cell cultures and across experiments depicted in different figures, the results are expressed as numbers of antibody secreting cells (ASC) normalized to 10^6^ cultured cells assayed by ELISPOT.

To identify B-cells engaged into BCR-specific interactions with influenza HA we first tried to stain PBMCs with monoclonal antibodies against the B-cell marker CD20 and the B-cell memory marker CD27 mixed with a recombinant H1 bait (rH1), or with human serum albumin (HSA), both conjugated with the Alexa-488 fluorochrome (A488). When stained with rHA, PBMCs gated on live singlets (Fig. 1A) showed a high and diffuse fluorescent signal on both B and non B-cells, while staining with HSA-A488 only gave background signal (Fig. 1B).

Human influenza HA is known to bind α2,6 sialic-acid residues [16], which are expressed on human leukocytes and are particularly abundant on B lymphocytes [17]–[18]. We tried to block this interaction by adding a 100-fold molar excess of soluble sialopentasaccharides containing α2,6-linkage to the staining solution. While this prevented indiscriminate binding of rH1 to most leucocytes, it was not sufficient to block rH1 binding to all B lymphocytes (Fig. 1B). Little, or no improvement was observed in experiments in which we pre-incubated PBMCs with compounds known to bind to α2,6-sialic-acid residues with high affinity, such as α-fetuin, or the *Sambucus nigra* lectin [19] (data not shown).

We then evaluated the possibility of removing sialic-acid residues from PBMC surfaces, or covering them with vaccine subunit mono-bulk antigens, purified from an influenza A subtype mismatched to that of the fluorescent HA bait. Equal numbers of PBMCs from the same donor were incubated for 30 minutes either at 37°C with *Clostridium perfringens* type VIII neuraminidase (NA), or on ice with a vaccine mono-bulk H3N2 antigens (from A/Panama/2007/1999). Both PBMCs samples were then stained with anti-CD20, anti-CD27 and an A488-tagged rH1 bait. Following either treatment the frequency of H1-binding B-cells was greatly reduced (2.2% of total B-cells in the NA-treated sample and 0.5% in the H3N2-pre-saturated sample) and most of the H1^+^ B-cells expressed the CD27 B-cell memory marker (Fig. 1C).

Because these staining patterns were consistent with that expected for MBCs binding to H1 through BCR-specific interactions, we sorted H1^+^ B-cells from both samples to verify their specificity by ELISPOT. To induce resting B-cells to differentiate into antibody secreting cells (ASC), the two sorted H1^+^ B-cell populations were cultured with CpG and IL-2, in the presence of autologous CD20-negative (CD20^neg^) feeder cells, in a ratio of 1∶200 to achieve a cell density of at least 10^6^ cell/ml. To exclude interference from *ex vivo* activated H1-specific plasmablasts putatively included among CD20^neg^ cells, an aliquot of CD20^neg^ cells was also mixed with H1-negative (H1^neg^) B cells sorted from the NA-treated sample and placed in culture with CpG and IL-2. Unsorted PBMCs were also activated in the same way to assess the starting frequency of H1^+^ ASC precursors. The results from these experiments showed that the two H1^+^ B-cell populations generated comparable numbers of ASC producing H1-specific IgG and at enriched frequencies as compared to unsorted PBMCs (Fig. 1D). The frequency of ASC producing H1-specific IgG detectable in cultures of CD20^neg^ cells mixed with H1^neg^ B cells was instead greatly reduced as compared to unsorted PBMC, showing that the contribution from H1-specific IgG ASC eventually generated from *ex vivo* activated CD20^neg^ plasmablasts was negligible. However, 95% of total IgG producing ASC generated from H1^+^ B-cells sorted from H3N2-pretreated PBMCs produced H1-specific antibodies; conversely, only 19% of IgG-ASC generated from H1^+^B-cells sorted following neuraminidase treatment were H1-specific (Fig. 1D).

These results were confirmed in 2 other experiments in which the proportion of H1-specific IgG-ASC generated from H1^+^ B-cells sorted from H3N2-, or NA-pretreated PBMCs accounted for 85–100% and 19–23% of total IgG-ASC, respectively.

Based on these results, we concluded that blocking BCR-independent binding sites with mismatched influenza subunit antigens increases the specificity of identifying H1^+^ B-cells and decided to utilize this staining approach in the subsequent experiments.

### Sensitivity and robustness of flow-cytometric analysis of HA-specific B-cell frequencies in *ex vivo* PBMCs

**Figure 2:**
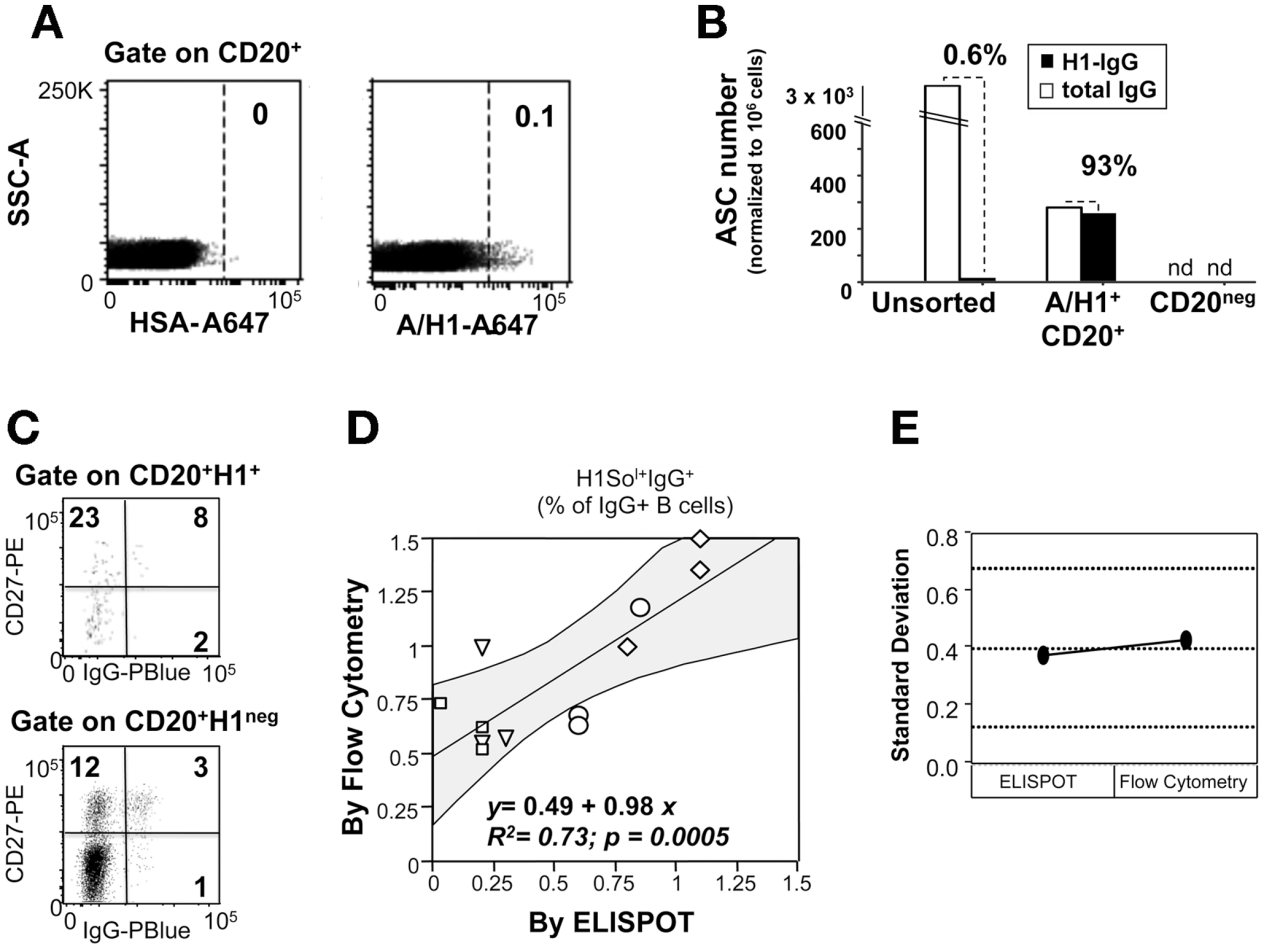
H1^+^ IgG^+^ MBCs frequencies measured by flow-cytometry and by ELISPOT correlated linearly. **A–B.** Specificity of the staining with the rH1 bait from A/Solomon Island/3/06. PBMCs (1.6×10^8^) from anonymous blood donors were stained with Live/Dead, incubated with an H3N2 mono-bulk vaccine subunit (from A/Panama/2007/1999), and then stained with Alexa647-conjugated HSA (6×10^7^), or Alexa647-conjugated rH1 (1×10^8^), and with an antiCD20 mAb. **A.** Binding pattern of HSA (A647-HSA; left panel) and of rH1 (A647-rH1; right panel) in the CD20^+^ B-cell gates. **B.** H1^+^ B-cells identified in A were sorted (n = 8215), mixed with autologous CD20^neg^ cells in the ratio of 1∶50 and activated with CpG and IL-2 for 5 days *in vitro*. Unsorted PBMC and CD20^neg^ cells were also cultured in the same manner as controls. After 5 days, equal numbers of cultured cells were harvested and assayed by ELISPOT for numbers of cells secreting IgG and IgG specific for H1N1 (from A/Solomon Island/3/06). Results are expressed as number of antibody secreting cells (ASC) normalized to 10^6^ cultured cells assayed by ELISPOT. Nd indicates undetectable ASC. **C.** Distribution of IgG^+^ B-cells among H1^neg^ and H1^+^ B cells expressing or not the CD27 B cell memory marker; shown is one representative subject. **D.** Replicates of frozen PBMCs from 4 anonymous blood donors were assayed by conventional ELISPOT, or incubated with an H3N2 mono-bulk vaccine subunit and stained with rH1, and anti-CD20 plus anti-human IgG antibodies. The scatter plot depicts paired values of H1^+^ IgG^+^ B-cell frequencies measured by flow-cytometry (y-axis) and by ELISPOT (x-axis) across three different experimental sessions. Shown are: the regression line with the related 95% confidence interval (gray areas), slope, intercepts, R^2^ and p-value. **E.** Variability plot showing mean standard deviations of the measurements done by ELISPOT and flow-cytometry. The three dotted lines mark the grand mean and the upper and lower control limits.

To obtain insights into the sensitivity and robustness of this approach, the frequencies of resting HA-specific IgG MBCs were measured both by flow-cytometry and by B-cell ELISPOT in PBMCs from 4 donors and in three different experimental sessions. For flow-cytometric analysis we used rH1 from the A/Solomon Island/03/2006 strain. The specificity of the H1^+^ B-cell subset was verified by ELISPOT following activation of sorted H1^+^ B-cells with CpG, IL-2 and autologous CD20^neg^ B cells for 5 days (Fig. 2 A and B) using PBMC from a fifth donor. The capture antigen used in the ELISPOT assay was the H1N1 mono-bulk subunit from the same vaccine strain. To assess the frequency of H1^+^ B cells expressing the IgG isotype among total H1^+^ B cells detected in each of the four donors, an anti-IgG mAb was added to the staining protocol (figure 2 C).

The results of these comparisons showed that the frequencies of H1-specific IgG MBCs detected by flow-cytometry and by ELISPOT were linearly correlated and that the correlation was statistically significant (Fig. 2D; R^2^ = 0.73; p = 0.0005). In addition, the variability of H1-specific IgG MBC frequencies measured by either method was comparable (Fig. 2E).

These results support the notion that our staining protocol can be applied for measuring HA^+^ MBC frequencies in *ex vivo* PBMCs samples, with comparable sensitivity and robustness to conventional ELISPOT assays.

### Simultaneous identification of B-cells specific for HA from A and B influenza strains in *ex vivo* PBMCs

**Figure 3:**
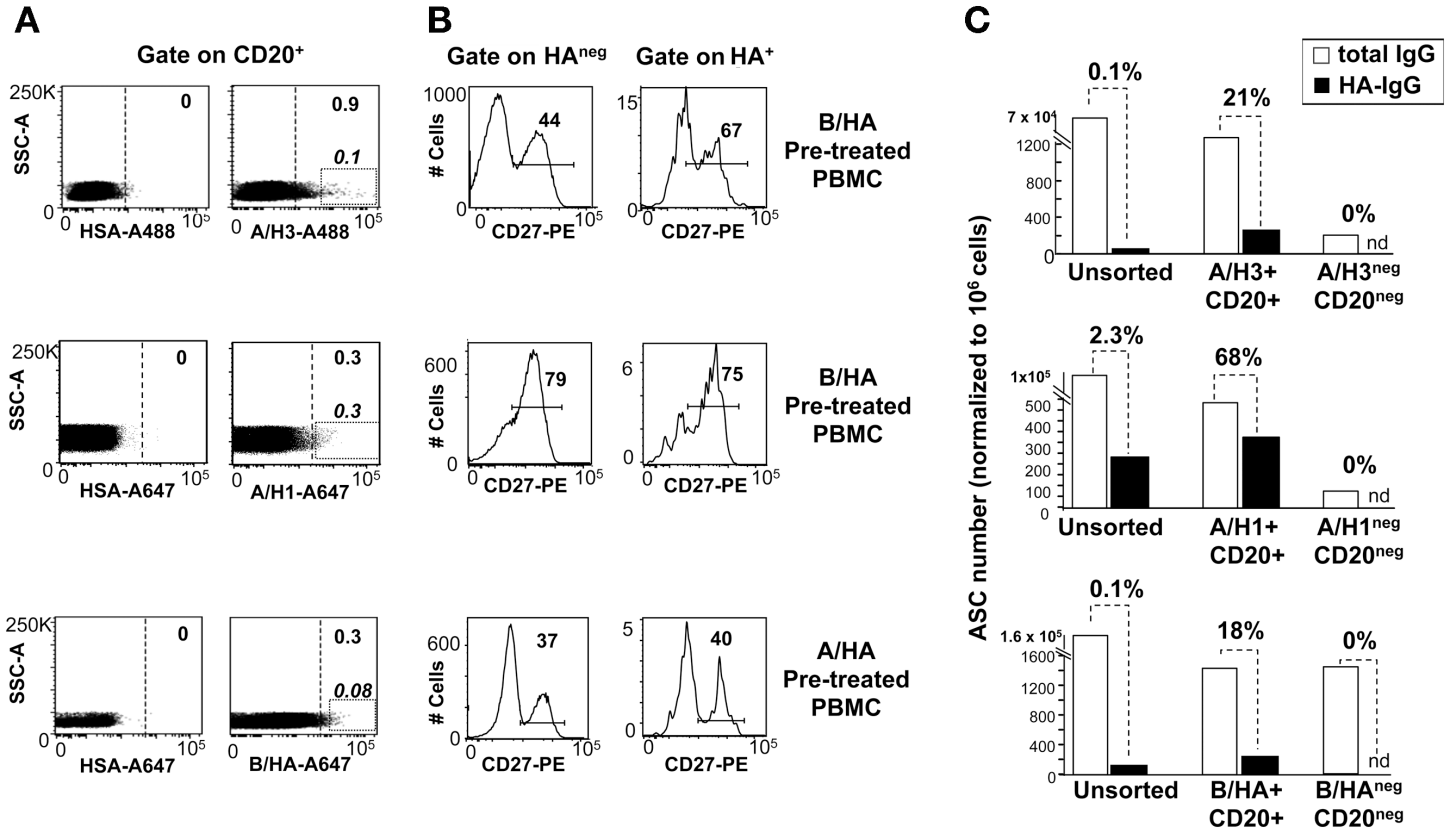
Identification of B lymphocytes specific for HA from A and B influenza strains in *ex vivo* PBMCs samples. PBMCs from different anonymous blood donors were pre-incubated with vaccine mono-bulk subunits from the B/Brisbane/60/2008 (B/HA pretreatment), or the H3N2 A/Panama/2007/1999 strain (A/HA pretreatment) and then stained HSA, rH3 (from A/Brisbane/10/2007), rH1 (from A/California/07/2009), or B/HA (from B/Brisbane/60/2008), as indicated. **A.** Staining pattern observed on CD20^+^ cells in PBMCs stained with the different rHA bait. The rectangular gates identify brilliant HA+ B-cells; the dotted vertical lines mark the gates used to sort HA^+^ B-cells for the ELISPOT assays. **B.** Expression of the CD27 memory marker on HA^+^ and HA^neg^ B cells identified based on the sorting gates. **C.** H3^+^ (n = 15,234), H1^+^ (n = 6482) and B/HA^+^ (n = 26,803) B-cells identified in A were sorted, mixed with autologous CD20^neg^ cells (in the ratio of 1∶20, 1∶100 and 1∶33) and activated with CpG and IL-2 for 5 days *in vitro*. Unsorted PBMCs and CD20^neg^ cells mixed with HA^neg^ B cells were also cultured in the same manner, as controls. After 5 days cultured cells were harvested and assayed by ELISPOT for the number of cells secreting IgG and IgG specific for mono-bulk subunits from the vaccine strain homologous to the sorting bait. Results are expressed as numbers of antibody secreting cells (ASC) normalized to 10^6^ cultured cells assayed by ELISPOT. Nd indicates undetectable ASC.

Next we assessed whether this approach was suitable to identify B-cells specific for HA from different influenza A subtypes, or from a type B influenza strain. PBMCs from two anonymous blood donors were pre-incubated with mono-bulk antigens from an influenza B vaccine strain and then stained with rHA from either A/H1N1 or A/H3N2 subtypes. Alternatively, PBMCs from a third donor were first pre-incubated with mono-bulk antigens from an influenza A/H3N2 subtype and then stained with B/rHA. B-cells putatively engaged in BCR-dependent binding to the rHA antigens were identified in each sample. Since applying a stringent gate only on bright H3+ or B/HA+ cells (rectangles in left panels of Fig. 3A) would have not permitted to obtain sufficient numbers of cells for ELISPOT tests, the sorting gates were set with lower stringency (dashed lines in left panels of Fig. 3A). In each sample HA^+^ B-cells distributed across memory (CD20^+^CD27^+^) and putatively naïve (CD20^+^CD27^−^) B cells in comparable manner to HA^neg^ B cells (Fig. 3B). All the HA^+^ B-cell populations were sorted and activated *in vitro* with CpG, IL-2 and autologous CD20^neg^ B cells, to verify the specificity of the staining by ELISPOT. Unsorted PBMC, as well as autologous CD20^neg^ B cells mixed with HA^neg^ B cells were also placed in culture as controls and activated in the same manner. After 5 days in culture, all the sorted HA^+^ B-cell populations were enriched in B-cells expressing IgG specific for the HA baits. Fig. 3C depicts the results from a representative experiment where the fold enrichments in IgG-ASC specific for H3 (from A/Brisbane/10/07), H1 (from A/California/07/09) or B/HA (from B/Brisbane/60/08) were 210×, 29×, and 180×, respectively. No IgG-ASC specific for HA were detected in cultures of CD20^neg^ B cells and HA^neg^ B cells (Fig. 3C). In this experiment the highest frequency of ASC producing HA-specific IgG HA was observed in the cultures of H1^+^ B-cells (68% of total IgG producing ASC), for which the most stringent sorting gate was applied (Fig. 3 A and C, middle panels). Comparable results were obtained with PBMCs from other two donors (not shown).

**Figure 4:**
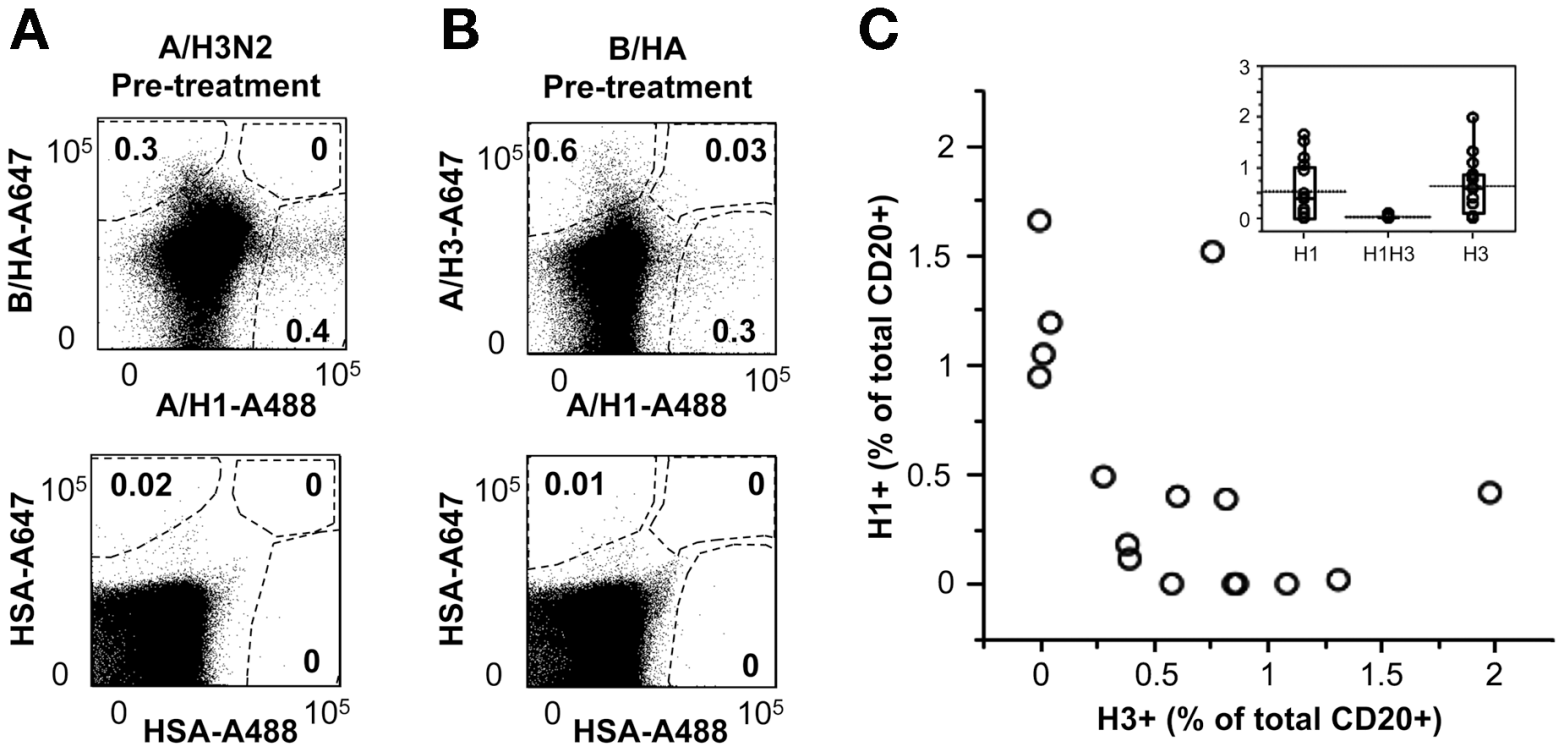
Simultaneous identification of B lymphocytes specific for HA from different influenza strains in *ex vivo* PBMCs samples. **A.** PBMCs from an anonymous blood donor were pre-incubated with subunits from either B/Brisbane/60/2008 or A/Panama/2007/1999 (H3N2), and then stained with anti-CD20 mAb, HSA conjugated with A488 and A647, with A647-rH3 (from A/Brisbane/10/2007) and A488-rH1 (from A/California/07/09), or with A647-rB/HA (from B/Brisbane/60/2008) and A488-rH1 (from A/California/07/09). The staining patterns observed in the CD20^+^ B-cell gate are shown. **B.** PBMCs from 16 anonymous blood donors were pre-saturated with B/Brisbane/60/2008 and then stained with anti-CD20 mAb, A647-rH3 (from A/Brisbane/10/2007) and A488-rH1 (from A/California/07/09). The scatter plot depicts paired values of H1^+^ (y-axis) and H3^+^ (x-axis) B-cells. The insert box plot depicts the distribution of H1^+^, H3^+^ and H1^+^H3^+^ B-cells in the same 16 donors. Mean values are indicated by dotted lines.

We then explored the possibility of combining two baits from different influenza strains to analyze B lymphocytes binding to either HA antigen in the same PBMC sample. Fig. 4 depicts the results obtained with PBMC from a single donor that were split into two tubes and either pre-incubated with a B/HA vaccine mono-bulk and stained with equal amounts of A647-rH3 and A488-rH1 (Fig. 4A), or pre-incubated with an A/H3N2 vaccine mono-bulk and stained with the A488-rH1 paired with an A647-B/rHA (Fig. 4B). H1^+^ B-cells were detected at comparable frequencies, irrespective of the type of the mono-bulk used as blocking agent (0.4 and 0.3% of total CD20^+^ B-cells), and segregated apart from B/HA^+^ or A/H3^+^ B-cells. The results obtained from the analysis of PBMCs from 16 different donors stained with A647-rH3 and A488-rH1 confirmed that the H1^+^ and H3^+^ B-cell subsets were largely non-overlapping (Fig. 4C). B-cells putatively cross-reactive with H1 and H3 were detected in 13 out of 16 subjects at frequencies 10 to 100-fold lower than those of B lymphocytes binding only to the H1 or the H3 bait (Fig. 4C, insert).

Overall, these results show that, by designing appropriate staining protocols, flow-cytometry can offer the unique opportunity to identify and sort single B-cells with restricted or cross-reactive binding capacity against different antigens.

### Molecular cloning of HA^+^ B-lymphocytes

Next, we assessed whether B-cells sorted based on HA binding are suitable for molecular cloning of IgV_H_V_L_ genes. To this aim, PBMCs from four blood donors were first incubated with B/HA vaccine antigens and then with an anti-CD20 mAb and the rH1-A647 and rH3–488 baits. B-cells that bound to H1 (H1^+^ B-cells) and B-cells that did not bind to either H1 or H3 (H1^neg^H3^neg^ B-cells) were sorted from two donors; from the other two donors we sorted H1^+^ B-cells and few cells binding to H3 (H3^+^ B-cells).

To clone paired V_H_V_L_ sequences we applied the single cell RT-PCR Ig-gene amplification protocol previously described [20]–[21]. The efficiency of molecular cloning ranged between 17% and 38%. Since these cells were obtained from frozen PBMCs, we couldn't directly compare these values to the 40–60% cloning efficiency obtained using freshly isolated PBMCs by the authors of the RT-nested PCR protocol [21]. Reassuringly, however, pairs of V_H_V_L_ sequences were obtained with comparable efficiencies from HA^+^ and HA^−^ B-cell subsets sorted from the same donor (38% for H1^+^ and 22% for H1^neg^H3^neg^ B-cells from donor #3, 17% for both H1^+^ and H1^neg^H3^neg^ B-cells from donor #4), suggesting that BCR-binding by rHA had no substantial impact on cell viability and mRNA degradation.

All arrays of sorted cells comprised single clones; only in one donor we found two H1^+^ clones that expressed identical V_H_V_L_ rearrangement with few different mutations (clones 53 and 87 from donor #2, Table S1).

**Figure 5:**
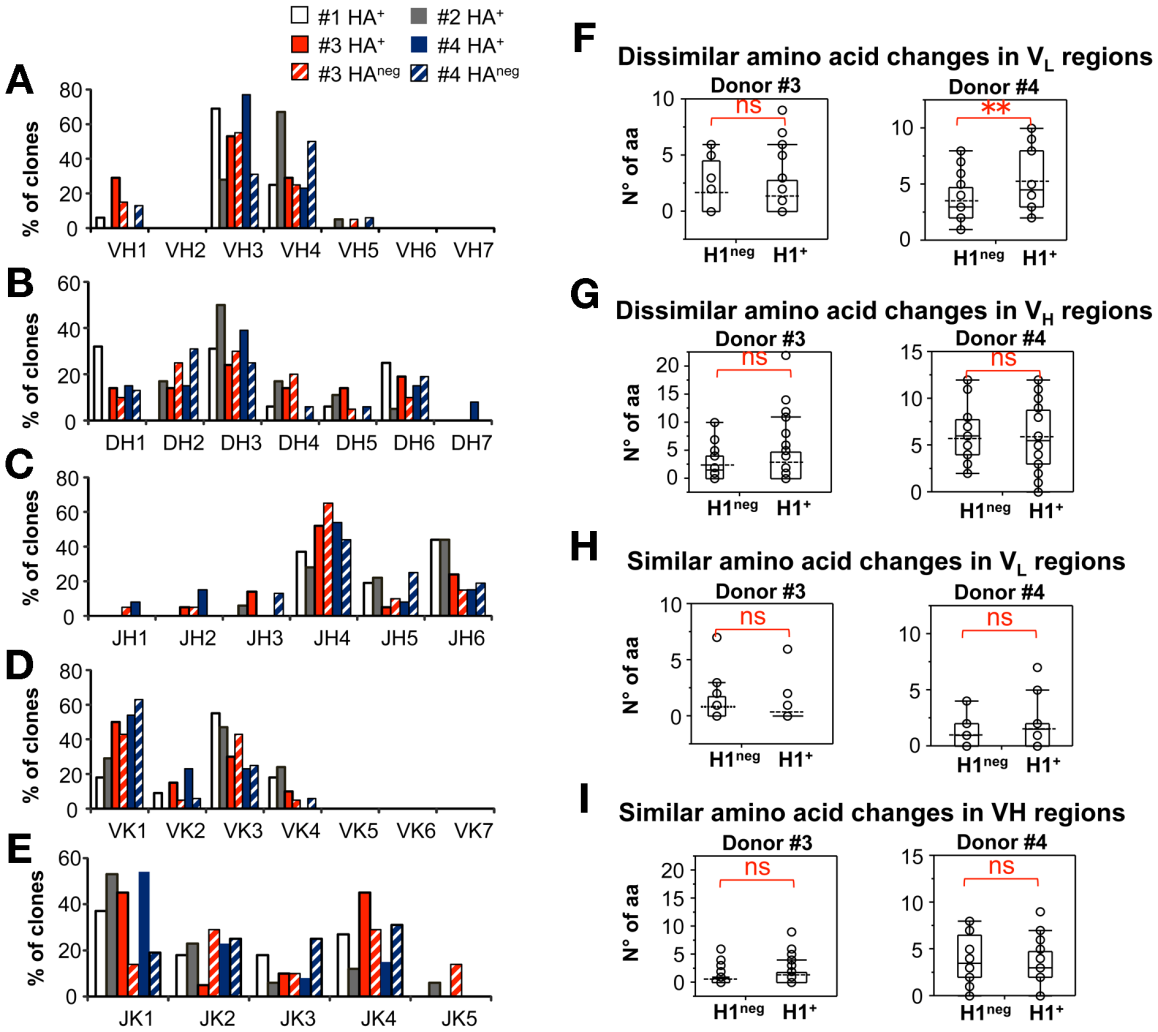
Molecular cloning of HA^+^ B lymphocytes. PBMCs from 4 anonymous blood bank donors were stained as in Figure 4B. Single H1^+^, H3^+^, or H1^neg^H3^neg^ CD20^+^ B-cells were sorted to perform molecular cloning and analysis of their paired V_H_V_L_ Ig regions as described in Material and Methods section. **A–E.** Distribution of V_H_ (A), D_H_ (B), J_H_ (C), V_k_ (D) and J_k_ (E) gene use across arrays of B-cells sorted from each donor (16 and 18 HA^+^ clones from donors #1 and #2; 35 HA^+^ and 20 HA^neg^ clones from donor 3; 16 HA^+^ and 16 HA^neg^ clones from donor #4. **F–I.** Number of mutations in H1^+^ and H1^neg^ CD20^+^ B-cells from donors #3 and #4, which cause dissimilar (F, H) or similar (G, I) amino acid substitutions in V_H_ (G,I) and V_L_ (F,H). NS and ** indicate not significant, or significant (p<0.036) difference between mean numbers of mutations by one-way Wilcoxon non-parametric test.

The distribution of V_H_ and V_L_ gene family use was similar across the different arrays of sorted B-cells, as well as across the four donors (Fig. 5). Use of V_H_3 segments was dominant in HA^+^ B-cells from three out of four donors; the second most represented family being V_H_4 and the remaining V_H_1 or V_H_5 (Fig. 5A). The D_H_3 family was most frequently used in HA^+^ B-cells from 3 donors, followed by D_H_2 and D_H_6 and at lower frequency by D_H_1, D_H_4 and D_H_5 (Fig. 5B). The most frequently expressed JH segments were from either the J_H_4 or the J_H_6 family, followed by J_H_5, J_H_3, J_H_2 and J_H_1 families (Fig. 5C). Similarly, genes from the V_K_1 and V_K_3 families were most frequently expressed in all arrays of clones and most of them were rearranged with J_K_1 or J_K_4 genes, less frequently with J_K_2, J_K_3 and J_K_5 genes (Fig. 5D and 5E).

Overall, no biases were found in the distribution of V_H_ and V_L_ gene families used by H1^+^ or H1^neg^H3^neg^ clones from the two donors analyzed in this manner (Fig. 5). An in depth analysis of V_L_ regions from H1^+^ and H1^neg^H3^neg^ B-cells showed that, in donor #4, these two B-cells subsets carried remarkable differences. As shown in Fig. 5F, V_L_ sequences cloned from H1^+^ B-cells of subject #4 displayed significantly higher numbers of mutations leading to dissimilar amino acid substitutions than V_L_ sequences from H1^−^H3^−^ B-cells (p<0.036, one-tail Wilcoxon non-parametric test). Conversely, no differences were found when comparing mutations leading to similar amino acid substitutions in V_L_ regions (Fig. 5H), suggesting that the H1^+^ and H1^neg^H3^neg^ B-cells could have evolved under different antigen-driven selection pressures. No differences were ever found between numbers of mutations leading to either dissimilar, or similar, amino acid substitutions in the V_H_ regions (Figs. 5 G and I).

In conclusion, these results show that the protocol developed to identify single HA-specific B-cells by flow-cytometry is applicable for molecular cloning and in-depth analysis of paired IgV_H_V_L_ genes.

### Analysis of qualitative and quantitative changes in HA+ B-cells induced by vaccination

Finally we asked whether the method we developed could be applied to analyze quantitative and phenotypic changes in the pool of HA-binding B cells induced by influenza vaccination. For this analysis we took advantage of the availability of PBMCs samples collected at baseline and at days 21 and 43 after vaccination from four volunteers enrolled in a study performed during the 2007/2008 northern hemisphere influenza season (E.F. et al. manuscript in preparation). These samples were incubated first with H3N2 mono-bulk antigens and then with an A647-rH1^+^ (from the 2007/2008 H1N1 vaccine strain A/Solomon Island/03/2006) and monoclonal antibodies against the CD20 B-cell marker, the CD27 memory marker and IgG.

**Figure 6:**
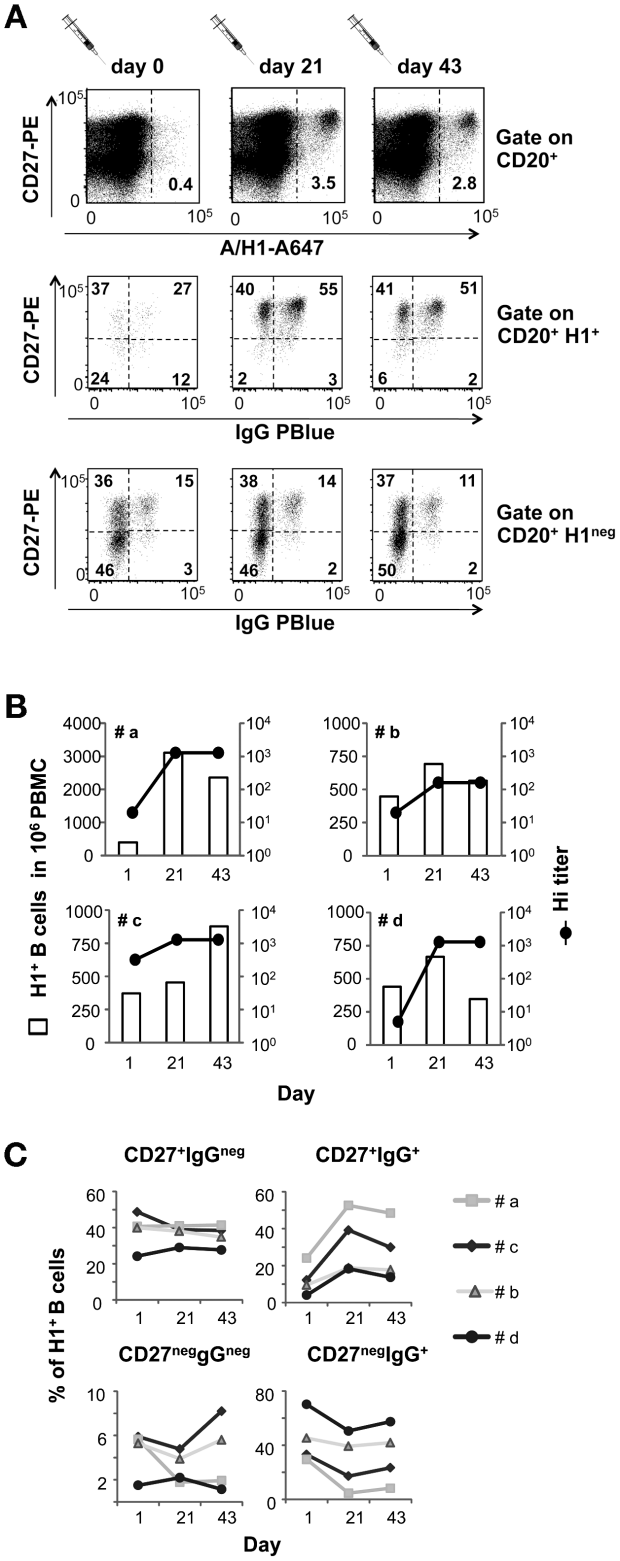
Vaccination induced changes in the pool of H1+ B-cells. PBMCs samples collected before (day 0) and at 3 and 6 weeks after vaccination from four seasonal influenza vaccinees, were pre-incubated with H3N2 subunit (from A/Panama/2007/1999) and then stained with rH1 A/Solomon Island/3/06) and mAbs anti-CD20, anti-CD27 and anti-human IgG. **A.** Dot plots gated on CD20^+^ B-cells showing the distribution of H1^+^ (middle panels) and H1^neg^ (bottom panels) B-cells from donor #a across: the mature memory (CD27^+^) and putatively naive (CD27^neg^) CD20+ B-cell subsets (upper panels); un-switched mature memory (CD27^+^IgG^neg^), IgG-switched mature (CD27^+^IgG^+^) and immature (CD27^neg^IgG^+^) memory B-cells. **B.** Numbers of circulating H1^+^ CD20^+^ B-cells in 4 vaccinees before and at 3 and 6 weeks after seasonal vaccination are overlaid with paired titers of antibodies inhibiting virus-induced hemmaglutination measured in their blood. The frequencies of H1^+^ B-cells are normalized according to the frequencies of CD20^+^ B-cells in 10^6^ PBMCs. **C.** Distribution of circulating H1^+^ and H1^neg^ B-cells across same subsets identified in **B** in all vaccinees.

At baseline, all subjects had measurable numbers of H1^+^ B-cells (Figs. 6A and 6B). Following vaccination, the frequencies of circulating H1^+^ MBCs cells increased from 1.5 to 8 fold over baseline values (Figs. 6A and 6B). In three out of four subjects the expansion of H1^+^ B-cell paralleled the rise of circulating antibodies capable of blocking hemagglutination. A delayed expansion of H1^+^ B-cells, measurable at day 43 was observed in subject c, which was the only subject with high antibodies against the H1N1 strain at baseline (Fig. 6B).

It is important to note that vaccination also induced remarkable changes in the distribution of H1^+^ B-cells across different MBCs subsets. At baseline, the majority of H1^+^ B-cells distributed across mature memory (CD27^+^) and immature IgG-switched memory cells (CD27^neg^IgG^+^) B-cells (Figs. 6A and 6C). By day 21, the proportion of H1^+^ B-cells expressing both the CD27 memory marker and an IgG-switched immunoglobulin receptor increased in all subjects to values that were 2 to 4-fold higher than those observed at baseline (Fig. 6C, upper right panel). The proportion of H1^+^ B-cells with naive (CD27^neg^IgG^neg^) or immature memory (CD27^neg^IgG^+^) phenotypes decreased in parallel (Fig. 6C, lower panels). Comparable changes were never observed in the population of H1^neg^ B-cells (Fig. 6A lower panels, and data not shown).

## Discussion

Our findings demonstrate that B-cells carrying immunglobulin receptors specific for type A or B influenza HA can be identified in *ex vivo* PBMCs by using fluorochrome-tagged rHA as antigenic baits and unlabeled mismatched mono-bulk vaccine subunit antigens to block BCR-independent binding. Circulating HA^+^ B cells identified in this manner in samples collected before and after vaccination can be further characterized for relative frequency and phenotype, as well as being sorted for further in depth analysis.

As antigenic bait, we used different recombinant full-length HA molecules, produced by Protein Sciences in the baculovirus-insect cell expression system. We opted for using rHA, instead of the influenza vaccine mono-bulk subunit preparations as bait for several reasons. First, the data available show that these soluble rHA preparations form trimers and higher order of structures, which retain hemagglutination activity and the expected antigenic properties of HAs [22]. In addition, rHA preparations do not contain other influenza proteins, while mono-bulk vaccine subunits contain HA and some NA. Finally, these rHA are commercially available and can be used by most laboratories.

There were two main obstacles to identifying, with high specificity, HA^+^ MBC circulating in human blood.

First, when added directly to untreated PBMCs, the rHA baits bind to all human blood leucocytes. This result is in apparent conflict with previous findings from the Baumgarth's lab supporting the use of flow-cytometry to analyze influenza HA specific B-cells [13]. Using lymph node cells from mice immunized with the influenza PR8 virus and soluble PR8 HA as bait, they did not observe BCR-independent binding and succeeded in obtaining an enriched population of HA-specific B-cells [13]. Such conflicting data can be reconciled by recent results showing that α2,6-N-acetyl sialic-acid residues, known to interact with high affinity with human influenza HA [16], are expressed at high levels on all human B lymphocytes, independently of their activation status, either as intrinsic component surface-expressed glycoproteins, or bound to the lectin domain of B-cell-restricted CD22 inhibitory receptors [23]. Conversely, most mouse B-cells express low levels of α2,6-N-acetyl sialic-acids on their surfaces, which transiently increase only in activated B-cells engaged in germinal center reactions [23]. Intriguingly, Baumgarth's group reported that close to 90% of the HA^+^ B-cells identified in PR8 immunized mice expressed surface markers characteristic of germinal center B-cells [13].

In line with the hypothesis that multimeric interactions between rHA and CD22-linked α2,6- N-acetyl sialic-acids could be responsible of the diffuse binding of HA to human B-cells, the addition of short α2,6-sialylated pentasaccharides to the staining solution displaced rH1 from most human leukocytes but not from B-cells. Conversely, rH1 binding to human B lymphocytes was blocked when PBMCs were stained following a pre-incubation step with either *C. perfringens* NA, which cleaves α-2,3- α-2,6- and α-2,8-linked sialic-acids, or with H3N2 human influenza mono-bulk antigens, which are known for preferential binding to α-2,6-sialic-acids. Of note, H1^+^ B-cells isolated from NA-treated PBMCs still contained a large number of cells producing IgG of unknown specificity. Because NA is less than 50% active at the pH and temperature conditions that preserve cell viability, the inefficiency of preventing non-specific HA binding by NA treatment was anticipated. In contrast, up to 95% of H1^+^ B-cells sorted from H3N2-preated PBMCs were confirmed as HA-specific by ELISPOT, supporting the use of this approach to prevent BCR-non-specific binding of fluorochrome-labeled rHA antigens.

The second obstacle to specific sorting of MBCs with HA baits was that the specificity of the HA^+^ B-cell population was influenced by the stringency of the gates applied in the analysis. Because human beings are exposed to a multiplicity of different antigenic challenges, finding a true universal negative control appears impossible. The use of autologous proteins, like HSA, is helpful to set the lowest gate for BCR-independent recognition. However, the use of such controls is clearly insufficient when dealing with pathogen receptor antigens, like HA, capable of multimeric interactions with their ligands. In our experience, the mean fluorescence intensity of HSA-stained B-cells is often substantially lower than that of *bona fide* HA^−^ B-cells stained with labeled rHA. Thus, to identify HA^+^ B-cells with great specificity we set the gate on brilliant HA^+^ B-cells. Repeated measurements of H1^+^IgG^+^ B-cell frequencies in replicates of PBMC samples by this staining approach provided a series of values linearly correlated with those obtained by ELISPOT assays, and with similar range of variability across different experimental sessions. Clearly, a robust standardization of the gating strategy would require parallel flow cytometric and ELISPOT analyses of large number of samples across different laboratories. It is encouraging, however, that the results obtained by analyzing PBMC samples from influenza vaccinees confirmed the adequacy of our gating approach to monitor quantitative and qualitative changes in HA-specific B-cell subsets induced by vaccination.

Quantitative analysis of MBCs before and after infection or vaccination has been so far performed by B-cell ELISPOT, or limiting dilution assays [24], [25]. These assays proved useful at gaining knowledge of the kinetic and the magnitude of MBC responses to influenza strains that did or did not circulate in the human population [3], [12], [24], [25]. The major limitation of these assays is that they are not suitable for functional and molecular analysis of the antigen-specific repertoire at the single cell level *ex vivo*.

We showed here that by combining rHA baits and monoclonal antibodies against surface B-cell markers our method is also suitable to analyze changes in the distribution of HA^+^ B-cells across MBCs subsets at different maturation stages [26] including in subjects who eventually did not experience a substantial increase in total number of HA^+^ MBCs. For our experiments we choose to restrict the analysis to HA^+^ B cells that expressed (or not) an IgG-switched BcR. A most in depth investigation of HA^+^ B cells expressing IgM or IgA receptors is clearly feasible by adding appropriate mAbs in the staining, as shown in Figure S1.

Overall, our observations provide strong support to the feasibility of standardizing flow-cytometry-based assays for monitoring directly in PBMC samples *ex vivo* quantitative and phenotypic changes induced in the repertoire of HA^+^ B-cells by influenza infection or vaccination. Moreover, the staining approach presented here can be used to sort arrays of single B-cells with different HA-binding specificities and to perform molecular cloning and in depth analysis of the V_H_V_L_Ig repertoires, as it is currently done with short-lived plasmablasts circulating early after antigenic challenge.

The possibility of combining two different HA baits in the same tube offers the opportunity of identifying rare circulating MBCs that cross-react between multiple influenza strains. Confirming the specificity of H3^+^H1^+^ B-cells identified in some of our samples was not feasible because the informed consents signed by the anonymous blood donors did not include producing monoclonal antibodies from their cells. In addition, the number of H3^+^H1^+^ B-cells in these samples was too low to apply an ELISPOT assay to verify their specificity. Nevertheless the results obtained by flow-cytometric analysis showed that the B-cells binding to A/H1 segregated completely from those binding the B/HA bait, consistent with the low amino acid sequence homology between these HA molecules and the absence of serological cross-reactivity between type A and B influenza strains [27]–[28]. Conversely, and in line with recent findings reported by multiple groups who used most complex B-cell cloning procedures [15], [29], in several PBMC samples we identified extremely low frequencies of B-cells putatively cross-reactive to group 1 (H1) and 2 (H3) A influenza strains.

A major limitation of our approach is that MCBs that cross-react between the blocking HA and the bait HA will be missed. Thus, the rare MBCs cross-reactive between type A H1 and H3 and B HA molecules as those recently discovered [14] would not be present in the sorted arrays of brightly stained MBCs unless they have extremely higher binding affinity to the fluorochrome-tagged HA baits as compared to the HA subunit used to block non specific binding.

Because human beings are exposed to annually changing antigenic variants of influenza HA, a deeper understanding of the protective potential of pre-existing MBCs repertoires is key to develop new and broadly protective vaccines. The approach reported here for direct analysis of HA-specific MBCs in *ex vivo* PBMC complements available methods to identify single plasmablasts, making possible to analyze all HA-specific B cell subsets circulating in the blood before and after vaccination. This will permit to verify the actual contribution of pre-existing MBCs in the generation of early plasmablasts and MBC expressing refined antibody specificities after infection or vaccination against novel (seasonal or pandemic) influenza viruses. With this complete set of tools longitudinal analyses of IgV_H_V_L_Ig phenotypically characterized repertoires of plasmablasts and HA^+^ MBCs from defined cohorts of subjects by deep sequencing will become possible, therefore allowing more rapid investigation of the *in vivo* B-cells dynamics driving the evolution of broadly cross-protective antibody responses against drifted influenza viruses. Greater understanding of these dynamics can aid the design of best vaccines for all ages and health conditions.

## Materials and Methods

### Human Blood samples and Ethics Statement

Leukopacs from anonymous healthy blood bank donors were collected during 2010–2011 season after written informed consent was provided and ethical approval granted by the Institutional Review Board of the Empoli City Hospital, the Comitato Etico Locale Azienda 11 Empoli. The history of influenza infection or vaccination of blood bank donors was unknown. Ethical approval for collecting blood and performing B-cell analyses from volunteers, enrolled across 2008 and 2009, in a Novartis' sponsored clinical study with trivalent seasonal influenza vaccine given with or without avian H5N1 vaccine formulated with MF59 adjuvant (ClinicalTrials.gov Identifier: NCT00620815), was granted by the Ethical Committee of the medical faculty of Ludwig-Maximilians-University Munich. All clinical investigations have been conducted according to the principles expressed in the Declaration of Helsinki. The results of this clinical study will be the subject of a forthcoming paper (Fragapane E. et al. unpublished data). PBMCs were isolated by conventional centrifugation over a Ficoll gradient, frozen and stored in liquid nitrogen as previously described [12]. All subjects provided written informed consent. The informed consents did not include permission for producing monoclonal antibodies from donors' blood cells.

### Conjugation of HA with fluorochromes

Recombinant HA (Protein Science) and HSA (Sigma-Aldrich) molecules were chemically labeled with Alexa Fluor 488 or Alexa Fluor 647 carboxylic acid succinimidyl ester (Molecular Probes, Invitrogen) following the manufacturer's instructions. Each protein antigen was incubated with the dye at a molar ratio of 1∶10 for 1 hour at room temperature and then loaded into a Zeba desalting spin column (Thermo Scientific) to remove unbound dye. The degree of labeling was determined following the manufacturer's instructions, by measuring the absorbance of conjugated protein at the relevant wavelength for each fluorochrome by spectrophotometry. Protein concentrations were calculated by the BCA Protein Assay (Pierce, Thermo Scientific); the protein integrity was analyzed by SDS-PAGE.

### Flow-cytometry

Frozen PBMCs were thawed at 37°C in PBS containing 2.5 mM EDTA and 20 µg/mL DNAse (Sigma Aldrich). Samples were stained in tubes, each containing 10^7^ PBMC, according to different protocols: i) For pre-saturation with mono-bulk vaccine antigens, PBMCs were first stained with Live/Dead Aqua (Invitrogen) diluted 1∶500 in 100 µl, for 20 min in the dark. Then 50 µl of PBS containing 20% rabbit serum were added for further 20 min at 4°C to saturate Fc receptors. After two washes in 2 ml of PBS, PBMCs pellets were dissolved in 10 µl of PBS containing 30 µg/ml of vaccine subunit antigens (or 60 µg/ml in case of staining with 2 antigen baits) and incubated at 4°C in PBS. After 15 min, 10 µl of PBS containing 30 µg/ml (approximately 4 micromoles) of each fluorochrome-conjugated rHA bait were added, together with 50 µl of a cocktail of pre-titrated amounts of anti-CD20 PrCPCy5.5 (Becton Dickinson, clone L27), anti-CD27 PE (Becton Dickinson, clone L128) and anti-hIgG PacificBlue (Jackson ImmunoRes) monoclonal antibodies 1% FBS for 1 hour at 4°C. After two washes with 2 ml of 1% FBS/PBS cells were diluted in 1 ml of 5 mM EDTA/PBS and acquired with the Canto II analyzer (Becton Dickinson, Pharmingen, San Diego, CA). ii) For neuraminidase pre-treatment, PBMCs pellets were diluted in 50 µl of PBS containing 0.1 to 5 M Type VIII neuraminidase from *Clostridium perfringens* (which cleaves α-2,3- α-2,6- and α-2,8-linked sialic-acid residues. # N5631 Sigma Aldrich), incubated for 30 min at 37°C and then washed 3 times with PBS. PBMCs were then stained as in i), but omitting the pre-incubation step with mono-bulk influenza antigens. iii) To saturate rHA binding sites for α2,6 linked sialic-acid residues, soluble sialopentasaccarides containing α2,6 linkage (provided by Dr. Paolo Costantino, Novartis VD) were incubated at 100-fold molar excess with labeled-rHA for 15 min at 4°C. PBMCs were then stained as in i), but omitting the pre-incubation step with mono-bulk influenza antigens. iv) For pre-incubation with the Sambucus Nigra lectin (Vector Laboratories), PBMCs were stained as in i) adding as saturation agent 100 µl of a solution of PBS containing 10 to 100 µg/ml of the lectin for 15 min at 4°C, instead of mono-bulk influenza antigens. Flow-cytometric analysis was performed using the FlowJo software (Treestar Inc.). For antigen-specific B-cell sorting, PBMCs from the same donor stained at 10^7^/tube were then pooled, diluted in 1 ml of 5 mM EDTA/PBS and filtered through 30 µm cup filcon (Becton Dickinson). Samples were maintained on ice until sorting. Sorting was performed in high purity mode by a FACS Aria instrument (BD Biosciences, San Jose, CA) equipped with the BD FACSDiva v6.1.3 software using a 70 µm nozzle operating at 70 psi. Sorting gates were established in order to isolate brilliant HA^+^ CD20^+^ cells, unless differently specified. HA negative (HA^neg^) B cells and CD20 negative (CD20^neg^) cells were also sorted as negative controls or feeders for HA^+^ and HA^neg^ B-cell cultures, respectively.

### ELISPOT

In order to induce resting B-cells to differentiate into antibody secreting cells, unsorted PBMCs HA^+^, or HA^neg^ B-cells were cultured *in vitro* in 0.2 ml of complete medium (RPMI with 100 units/mL penicillin, 100 µg/mL of streptomycin, 2 mM L-glutamine, 1 mM sodium pyruvate, and 0.1 mM not essential amino acids, 5%FBS; Invitrogen) containing 2.5 µg/ml of CpG (PRIMM, Milan, Italy) and 1000 U/ml of IL-2 (Novartis). CD20- B-cells were added as feeders to HA^+^ B-cell cultures in variable ratios (from 1∶30 to 1∶200) to achieve a cell density of at least 10^6^/ml. After 5 days, cultured cells were washed 4 times in PBS, counted, diluted to equal concentrations in complete medium and plated in complete medium (200 µl/well) in serial 2-fold dilutions in duplicate-triplicate wells of ELISPOT plates pre-coated with HSA, influenza antigens or an anti-human IgG antibody. ELISPOT plates (MultiScreen HTS™ HA, Millipore) were coated over night at 4°C with 100 µg/ml of PBS containing 2.5 µg/ml of an anti-human IgG (BD Pharmingen), or 10 µg/ml of HSA (Sigma), H1N1 from A/California/07/2009 or A/Solomon Island/03/2006, or H3N2 from A/Brisbane/10/2007, or HA from B/Brisbane/60/2008. Following an overnight incubation at 37°C and 5% CO_2_, the ELISPOT plates were washed 4 times with PBS, then 4 times with 0.05% Tween20/PBS. Spots of antibody secreting cells were revealed with biotin-conjugated mouse anti human IgG (Southern Biotech) diluted in 4% BSA/PBS for 4 hours at 37°C, followed by horse radish peroxidase-conjugated Streptavidin (ENDOGEN). The reaction was then developed with 3-amino-9-ethylcarbazole (AEC, Sigma). Automated spot counting was performed using the UV Spot ELISPOT plate Analyzer (CTL) and the Immunospot software v5.09 (CTL).

### Single cell RT-PCR and sequence analysis of Ig variable regions

Single HA^+^ and HA^−^ CD20^+^ B-cells were sorted into ice-cold 96-well PCR plates pre-filled with 4 µl/well of 0.5× PBS containing 10 mM DTT (Invitrogen), 8 U RNasin (Promega), 0.4 U RNAse Inhibitor (QIAGEN). After sorting, plates were sealed, centrifuged and immediately transferred at −80°C. cDNA synthesis was performed adding 10.5 µl mix to the 96-well plate containing the sorted cells. The mix contains 150 ng of random hexamer primers (Roche), 0.5% v/v Igepal (SIGMA CA-630), 1 U RNAse Inhibitor (QIAGEN), 4 U RNAsin (Promega), 10 mM DTT (Invitrogen), 0.5 mM dNTP mix (Roche), 50 U SuperScript III Reverse Transcriptase (Invitrogen), 5× Reverse Transcriptase Buffer (Invitrogen) diluted in nuclease free water (Invitrogen). The reaction was incubated at 25°C for 5′, at 50°C for 60′, and at 70°C for 15′. For the 1^st^ PCR round 3.7 µl of cDNA template were mixed with forward primer mixes specific for the leader region and reverse primers specific for either IgH, IgK and Igλ constant regions (20–21) in 3 different plates. For the 2^nd^ nested PCR round, 2.5 µl of the first round PCR product were mixed with nested forward and reverse primers mixes [20]–[21]. Both PCR reactions were performed in 96 well plates (Eppendorf) in a total volume of 25 µl/well of nuclease free water containing 1× GoTaq Hot Start Green Master Mix (Promega) and 0.2 µM of each primer or primer mix. Each round of PCR was performed for 50 cycles at 94°C for 30 seconds, 57°C (1^st^ PCR) or 59°C (2^nd^ PCR) for 30 seconds and 72°C for 55 seconds (1^st^ PCR) or 45 seconds (2^nd^ PCR). PCR products were separated on 2% agarose gels and analyzed for the presence of 350–400 nucleotides products. Positive PCR products were purified from the wells of the plate using the QIAquick PCR Purification Kit (QIAGEN). PCR products were sequenced with the ABI 3730xl 96-capillary DNA analyzer (Applied Biosystems), using the second round PCR forward primer mix for the heavy chain products, the PanVK primer for the kappa light chain product, the second round reverse primer for the lambda light chain products. Sequences of the IgH/L V region were analyzed by IMGT/V-QUEST system.

### Statistical analysis

A linear regression analysis was performed using the JMP 8.0.1 software to assess the presence of a linear correlation between frequency of H1^+^ IgG memory B-cells measured by flow-cytometry, or by conventional B-cell ELISPOT. Variability analysis was performed to compare the repeatability of the two assays. To avoid assuming that frequencies of amino acid replacements had a normal distribution, the mean frequencies of amino acid replacements between arrays of H1^+^ and H1^neg^ B-cells were analyzed by one-way Wilcoxon non-parametric test as implemented in the stats package of R version 2.14 (http://www.r-project.org/).

## Supporting Information

Figure S1 **Expression of IgM, IgA and IgG BcR among H1^+^ B-cells in steady state.** PBMCs from anonymous blood bank donors were pre-incubated with H3N2 subunit (from B/Brisbane/60/2008) and then stained with rH1 A/California/07/09 and mAbs anti-CD19, anti-CD27, anti-IgM and anti-IgG, or anti-IgA and anti-IgG. **A.** Dot plots gated on CD19^+^ B-cells showing the binding pattern of HSA or rH1 across mature memory (CD27^+^) and putatively naive (CD27^neg^) B-cells from donor #2. **B.** Dot plots showing the distribution of cells expressing IgA or IgG, or IgM BcR across CD27^+^ or CD27^neg^ H1^+^ and H1^neg^ B cells. **C.** Frequencies of IgA, IgM and IgG BcR across the same subsets identified in **B** in four different donors.
(TIF)

Table S1 **V_H_V_L_ rearrangements, mutation frequency and length of the CDR3 regions in individual HA^+^ B cell clones analyzed in** **figure 5** **.**
(XLSX)
