# Supplementary figures and images for: Ex Vivo Analysis of Human Memory B Lymphocytes Specific for A and B Influenza Hemagglutinin by Polychromatic Flow-Cytometry
Source: PLoS One. 2013 Aug 15;8(8):e70620. doi: 10.1371/journal.pone.0070620 (PMC3744578; doi:10.1371/journal.pone.0070620)

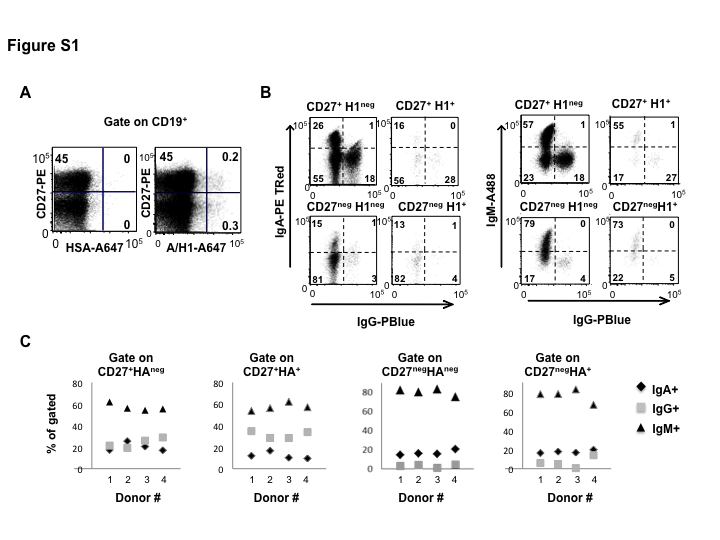

Supplement: Figure S1 — Expression of IgM, IgA and IgG BcR among H1+ B-cells in steady state. PBMCs from anonymous blood bank donors were pre-incubated with H3N2 subunit (from B/Brisbane/60/2008) and then stained with rH1 A/California/07/09 and mAbs anti-CD19, anti-CD27, anti-IgM and anti-IgG, or anti-IgA and anti-IgG. A. Dot plots gated on CD19+ B-cells showing the binding pattern of HSA or rH1 across mature memory (CD27+) and putatively naive (CD27neg) B-cells from donor #2. B. Dot plots showing the distribution of cells expressing IgA or IgG, or IgM BcR across CD27+ or CD27neg H1+ and H1neg B cells. C. Frequencies of IgA, IgM and IgG BcR across the same subsets identified in B in four different donors. (TIF) [file pone.0070620.s001.tif]
